# 20-Hydroxyeicosatetraenoic Acid (20-HETE) Modulates Canonical Transient Receptor Potential-6 (TRPC6) Channels in Podocytes

**DOI:** 10.3389/fphys.2016.00351

**Published:** 2016-08-31

**Authors:** Hila Roshanravan, Eun Y. Kim, Stuart E. Dryer

**Affiliations:** ^1^Department of Biology and Biochemistry, University of HoustonHouston, TX, USA; ^2^Division of Nephrology, Baylor College of MedicineHouston, TX, USA

**Keywords:** podocytes, calcium, FSGS, ion channels, eicosanoids, TRPC6

## Abstract

The arachidonic acid metabolite 20-hydroxyeicosatetraenoic acid (20-HETE) regulates renal function, including changes in glomerular function evoked during tubuloglomerular feedback (TGF). This study describes the cellular actions of 20-HETE on cultured podocytes, assessed by whole-cell recordings from cultured podocytes combined with pharmacological and cell-biological manipulations of cells. Bath superfusion of 20-HETE activates cationic currents that are blocked by the pan-TRP blocker SKF-96365 and by 50 μM La^3+^, and which are attenuated after siRNA knockdown of TRPC6 subunits. Similar currents are evoked by a membrane-permeable analog of diacylgycerol (OAG), but OAG does not occlude responses to maximally-activating concentrations of 20-HETE (20 μM). Exposure to 20-HETE also increased steady-state surface abundance of TRPC6 subunits in podocytes as assessed by cell-surface biotinylation assays, and increased cytosolic concentrations of reactive oxygen species (ROS). TRPC6 activation by 20-HETE was eliminated in cells pretreated with TEMPOL, a membrane-permeable superoxide dismutase mimic. Activation of TRPC6 by 20-HETE was also blocked when whole-cell recording pipettes contained GDP-βS, indicating a role for either small or heterotrimeric G proteins in the transduction cascade. Responses to 20-HETE were eliminated by siRNA knockdown of podocin, a protein that organizes NADPH oxidase complexes with TRPC6 subunits in this cell type. In summary, modulation of ionic channels in podocytes may contribute to glomerular actions of 20-HETE.

## Introduction

Podocytes are terminally differentiated multipolar cells that cover the outer surface of the glomerular capillary and form the final barriers for glomerular filtration. Changes in podocyte ultrastructure often occur in response to glomerular injuries and in a variety of disease processes. If injuries are sustained, and if sufficient numbers of podocytes detach or die, the entire nephron will be lost (Kim et al., 2001; Kriz and LeHir, 2005). Primary podocyte diseases have multiple etiologies. Adult-onset familial forms of focal and segmental glomerulosclerosis (FSGS) occur with an autosomal dominant mode of inheritance in patients with mutations in the gene encoding canonical transient receptor potential-6 (TRPC6) channels. Most of these mutations result in a gain of function when the encoded channels are assayed in heterologous expression systems (Reiser et al., 2005; Winn et al., 2005). Moreover, proteinuria and glomerulosclerosis were observed in mice over-expressing wild-type or mutant TRPC6 channels in podocytes (Krall et al., 2010) or in mice with podocyte-specific mutations in heterotrimeric G proteins that lead to constitutive activation of TRPC6 (Wang et al., 2015). Therefore, it is important to understand physiological signals that regulate the activity of TRPC6 channels in podocytes.

TRPC6 channels are Ca^2+^-permeable cationic channels that play a role in G protein-mediated signaling in diverse cell types. Within the kidney, TRPC6 channel subunits are expressed in mesangial cells and podocytes (Bouron et al., 2016; Ma et al., 2016), and probably also in renovascular myocytes such as those in the afferent arteriole (Salomonsson et al., 2010). Within podocytes, TRPC6 channels are located in the cholesterol-rich slit diaphragm domains of foot processes, as well as in major processes, and in the cell body (Reiser et al., 2005; Huber et al., 2006; Dryer and Reiser, 2010). TRPC6 channels in foot processes are components of large super-complexes that include podocin, nephrin, NADPH oxidases, phospholipases, and tyrosine kinases (Reiser et al., 2005; Huber et al., 2006; Kanda et al., 2011; Anderson et al., 2013; Kim et al., 2013; Kalwa et al., 2015; Rinschen et al., 2016).

A substantial body of evidence has suggested important roles for 20-hydroxyeicosatetraenoic acid (20-HETE) in renal function and dysfunction. 20-HETE is the ω-hydroxylated metabolite of arachidonic acid produced by a family of cytochrome P450 variants known as CYP4A and CYP4F (Roman, 2002). 20-HETE evokes vasoconstriction of various microvessels (Zhao et al., 2004) owing in part to increased Ca^2+^ influx into vascular myocytes (Zhao et al., 2004; Yaghi and Sims, 2005). In addition to its effects on renovascular smooth muscle, 20-HETE also modulates ion transport in the proximal tubule (Dos Santos et al., 2004) and thick ascending loop of Henle (Yu et al., 2007). While it is generally believed that 20-HETE can function as an intracellular second messenger underlying responses to vasoconstrictors and during renal vascular autoregulation (Hoopes et al., 2015), there is evidence that 20-HETE can be stored and released from pre-glomerular renal microvessels, potentially leading to effects on other renal cell types through paracrine signaling mechanisms (Carroll et al., 1997; Croft et al., 2000).

In vascular smooth muscle, 20-HETE has been reported to cause activation of TRPC6 channels and to act synergistically with exogenous diacylglycerol (DAG), the canonical lipid activator of TRPC6 (Inoue et al., 2009). This induces depolarization and activation of voltage-activated Ca^2+^ channels of myocytes leading to vasoconstriction and changes in renal blood flow, for example during tubuloglomerular feedback (TGF) (Ge et al., 2014). 20-HETE has also been implicated in communications between vascular endothelial cells and neighboring cells (Hilgers and De Mey, 2009). In this regard, recent studies have also demonstrated that TGF induces a wave of elevated cytosolic Ca^2+^ in extraglomerular mesangial cells that propagates from cell to cell, eventually leading to Ca^2+^ influx in the most distant podocytes and contraction of the entire glomerular tuft (Peti-Peterdi, 2006). Increases in podocyte intracellular Ca^2+^ also occur in response to focal injuries, and spread from one podocyte to several others (Burford et al., 2014).

In the present study we have examined modulatory effects of 20-HETE on the activity of TRPC6 channels in podocytes. In contrast to vascular smooth muscle or many mesangial cells (Yu et al., 1989; Hall et al., 2000), podocytes are non-excitable cells (Dryer and Reiser, 2010), and Ca^2+^ influx is primarily mediated by receptor-activated channels in the TRPC family (Ilatovskaya et al., 2014). We now report that 20-HETE exposure increases the steady-state surface expression of TRPC6 subunits and increases cationic currents through TRPC6 channels in podocytes. The actions of 20-HETE on podocyte TRPC6 channels appear to be indirect, as they require generation of reactive oxygen species (ROS) and activation of GTPases. These responses may also depend on the local membrane environment as they are eliminated following podocin knock-down.

## Materials and methods

### Drugs and reagents

The pan-TRP channel inhibitor 1-[2-(4-methoxyphenyl)]-2-[3-(4-meth-oxyphenyl)propoxy]ethyl-1H-imidazole hydrochloride (SKF-96365), the ROS scavenger 1-oxyl-2,2,6,6-tetramethyl-4-hydroxypiperidine (TEMPOL), and guanosine 5′-[β-thio]diphosphate (GDP-βS) were obtained from Sigma. 20-HETE and 1-oleoyl-2-acetyl-*sn*-glycerol (OAG) were purchased from Cayman Chemical Co.

### Podocyte cell culture, ROS assays, immunoblot analysis, and siRNA procedures

An immortalized mouse podocyte cell line (MPC-5) originally developed by Dr. Peter Mundel of Harvard Medical School was maintained at 33° as described previously on collagen-coated glass coverslips (Kim et al., 2008). Podocyte differentiation and expression of podocyte markers were induced by removal of γ-interferon and raising temperature to 37°C for 14 days. TRPC6 siRNA, podocin siRNA and non-targeting control siRNA were transfected into differentiated MPC-5 cells after isolation using Lipofectamine 3000™ (Invitrogen) in serum-reduced medium according to the manufacturer's directions (Anderson et al., 2013). Electrophysiological and biochemical analyses were carried out 24 h after transfection with siRNAs. Panels of siRNAs were purchased from Santa Cruz Biotechnology (Santa Cruz, CA) that target three different unique sequences within TRPC6 (catalog sc-42673) and podocin (sc-40860), and a non-targeting control siRNA (sc-37007). This vendor uses a proprietary system to develop siRNAs that target three different and unique 9-25 nucleotide portions of the transcript of interest. The oligonucleotides are supplied as an RNA duplex in solution.

The siRNA sequence of the antisense oligonucleotide strands that knock down TRPC6 are:

5′UUAACAUUGAGGGAAUGACtt3′
5′UAAUCUUCUGAGCUCCUUGtt3′
5′UUCUAAUGAGCUCUGCUAGtt3′

The antisense siRNA sequences used to knock down podocin are:

5′AUCUUGGGCAAUGCUCUUCtt3′
5′AGUUGCUUCAAGUUCUUGCtt3′
5′UUGUUUGCCAACUUGAUACtt3′

Immunoblot analyses and cell-surface biotinylation assays were carried out by standard methods as described in previous studies (Kim et al., 2013). Rabbit anti-TRPC6 was obtained from Alomone Laboratories (Jerusalem, Israel, catalog number ACC-017) and rabbit anti-podocin was purchased from Santa Cruz Biotechnology (catalog number sc-21009). The TRPC6 antibodies from Alomone Laboratories nearly always allow for ready detection of TRPC6. However, in our experience, different lots of the antibody vary considerably in their sensitivity and therefore in the number of cells needed to obtain a robust signal. The experiments in which electrophysiology was carried out after TRPC6 or podocin knockdown experiments were based on three different transfections on different days for each siRNA. Immunoblots were analyzed by densitometry using Image J™ software (Bethesda, MD). Graphs summarizing those data are plotted as mean ± SD, since this is the preferred method of showing data dispersion with a triplicate measures in biochemical experiments. Steady-state intracellular ROS levels of podocytes were estimated using the OxiSelect fluorometric assay (Cell Biolabs, San Diego, CA) according to the manufacturer's instructions. In this assay, a cell-permeable probe, 2′,7′-dichlorodihydrofluorescin diacetate is deacetylated by cytosolic enzymes to yield a non-fluorescent product that is trapped in the cytosol. In the presence of intracellular ROS, this probe is oxidized to a fluorescent product, 2′,7′-dichlorodihydrofluorescein (DCF), which is measured using a fluorescent microplate reader at excitation and emission wavelengths of 485 nm and 530 nm, respectively. These measurements were repeated in three different cell culture preparations exposed to 20-HETE or control medium.

### Electrophysiology

Methods for conventional whole-cell recordings from podocytes were carried out by standard methods that have been described in detail previously (Anderson et al., 2013, 2014; Roshanravan and Dryer, 2014). Recordings were made with an Axopatch 1D amplifier (Molecular Devices) and analyzed using pClamp™ *v*10 software (Molecular Devices). For analyses of currents through TRPC6, the bath solution contained 150 mM NaCl, 5.4 mM CsCl, 0.8 mM MgCl_2_, 5.4 mM CaCl_2_, and 10 mM HEPES, pH 7.4. Pipette solutions contained 10 mM NaCl, 125 mM CsCl, 6.2 mM MgCl_2_, 10 mM HEPES, and 10 mM EGTA, pH 7.2. The presence of Cs^+^ in bath and pipette solutions, and the absence of K^+^ in those solutions, precludes current flowing through KCa1.1 channels that are also expressed in these cells (Kim et al., 2008). The relatively high Ca^2+^ concentration in the bath solution markedly increases stability of whole cell recordings. In experiments on TRPC6, currents were periodically evoked by 2.5-s ramp voltage commands (−80 mV to +80 mV) from a holding potential of −40 mV. Whole cell current amplitudes were quantified at +80 mV. Recording pipettes in all experiments had resistances of 2–5 MΩ, and it was possible to maintain stable recordings while compensating up to 80% of this series resistance.

### Statistical analyses

Comparisons between multiple groups were carried out using one-way ANOVA followed by Tukey's HSD *post hoc* test using online packages at http://www.vassarstats.net Comparisons between two groups were carried out using Student's unpaired *t*-test using the same on-line resource. In all cases *P* < 0.05 was regarded as significant. Data from electrophysiological experiments, in which each group contained 6–12 cells, are presented as mean ± SEM. Data from biochemical experiments, all of which were repeated on three separate preparations of cells, are shown graphically as mean ± SD.

## Results

### 20-HETE increases currents through TRPC6 channels and increases abundance of TRPC6 subunits on the podocyte cell surface

**Figure 1:**
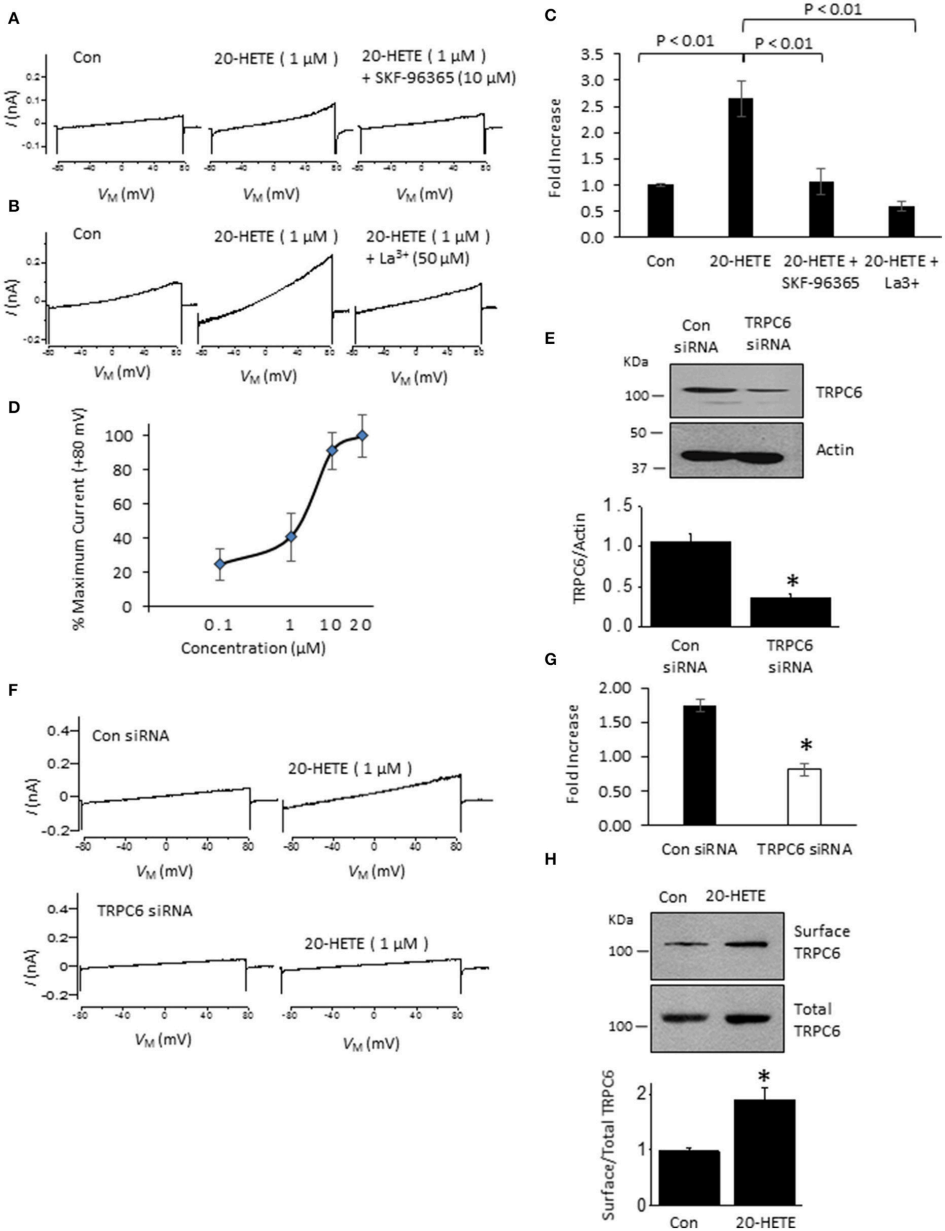
**Currents activated by 20-HETE in cultured podocytes. (A)** Examples of currents evoked by 2.5-s ramp voltage commands (−80 mV to +80 mV). Trace to the left shows currents shortly after making whole-cell contact. Middle trace is from the same cell 3 min after exposure to 1 μM 20-HETE, and trace to the right shows complete blockade of the current by SKF-96365. **(B)** Similar recording from a different cell showing complete block of 20-HETE-evoked current by 50 μM La^3+^. **(C)** Summary of results of this experiment (*n* = 10 cells per group). Data are presented as fold-increase over initial baseline current at +80 mV. The differences in means were calculated by Tukey's HSD *post hoc* test following one-way ANOVA. **(D)** Percentage of maximal current amplitudes at +80 mV plotted vs. concentration of 20-HETE. Note saturation of the response by 20 μM 20-HETE. **(E)** Immunoblot analysis showing effects of non-targeting siRNA (con) and siRNA targeting TRPC6 on total abundance of TRPC6. A typical immunoblot is shown above graphical summary of experiments from three different transfections. Asterisk indicates *P* < 0.01 (unpaired *t*-test). **(F)** Examples of responses to 1 μM 20-HETE in cells pre-treated with non-targeting (control) or TRPC6-targeting siRNA using procedures shown in E. Note lack of response to 20-HETE after TRPC6 knockdown. **(G)** Summary of the results of the experiment in panel F (*n* = 6 cells per group). Asterisk indicates *P* < 0.05 (unpaired *t*-test). **(H)** Cell-surface biotinylation assays show that exposure to 1 μM 20-HETE for 24 hr increases steady-state surface abundance of TRPC6. Typical blots are shown on top of graphical summary of three repetitions of this experiment. Asterisk indicates *P* < 0.05 (unpaired *t*-test).

**Figure 2:**
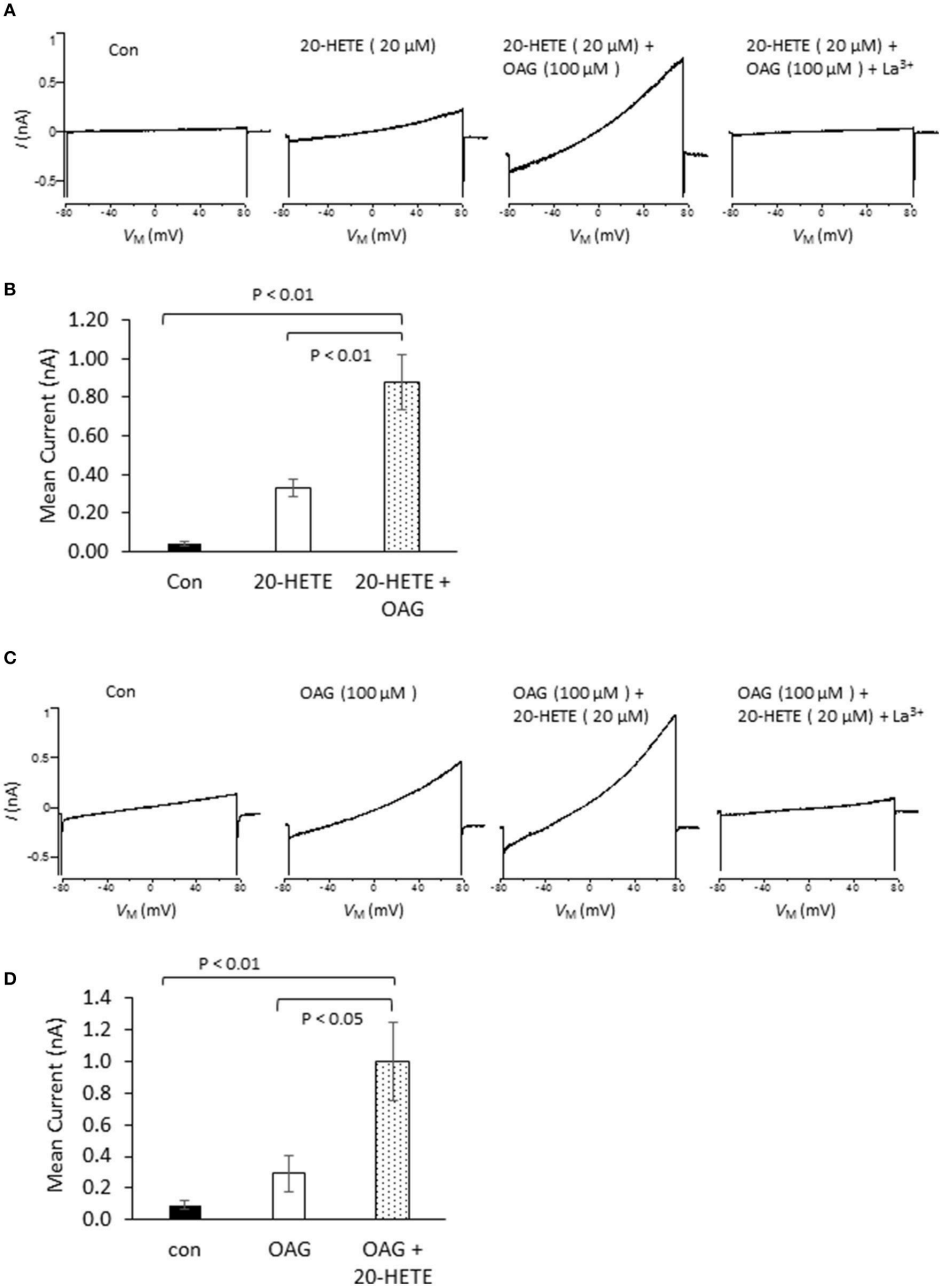
**Effects of 20-HETE in presence and absence of a membrane-permeable analog of diacylgycerol (OAG). (A)** Examples of currents from a single podocyte shortly after making whole-cell contact; after exposure to 20 μM 20-HETE; and then after 100 μM OAG was added to the superfusate. At the end of the experiment, 50 μM La^3+^ was added to the superfusate, which brought currents back to the original baseline. Note that OAG increased currents even in presence of a maximally active concentration of 20-HETE. **(B)** Summary of the results of this experiment (*n* = 5 cells per group). Data were analyzed by Tukey's HSD test following one-way ANOVA. **(C)** Similar experiment to the one shown in **(A)**, except that cells were first exposed to 100 μM OAG and then exposed to the combination of lipids. **(D)** Summary of results of the experiment shown in **(C)**. The responses to OAG and 20-HETE are approximately additive.

In initial experiments, we exposed differentiated immortalized mouse podocytes (MPC-5 cells) to 1 μM 20-HETE by gravity-fed bath superfusion during conventional whole-cell recordings in which we periodically applied ramp voltage commands (−80 mV to +80 mV over 2.5 s). About 1–2 min after the onset of 1 μM 20-HETE application, we observed an increase in an outwardly rectifying current observed during the voltage ramps. This current was eliminated by subsequent exposure to either 10 μM SKF-96365, a pan-TRP channel blocker (Figures 1A,C), or by 50 μM La^3+^, an ion that inhibits TRPC6 but not TRPC5 channels (Figures 1B,C) (Jung et al., 2003; Tian et al., 2010; Anderson et al., 2014; Roshanravan and Dryer, 2014). This last experiment was carried out because TRPC5 channels are expressed in podocytes (Tian et al., 2010), although as yet the factors that cause them to become active are not known. Activation of currents in podocytes occurred at micromolar concentrations of 20-HETE, with maximal effects occurring at a nominal concentration of 10–20 μM (Figure 1D). To further assess whether 20-HETE-induced cationic currents require TRPC6 channels, we transiently knocked down TRPC6 channels using siRNAs that target TRPC6 (Figure 1E). Control cells were transiently transfected with non-targeting siRNAs. We observed that 20-HETE activation of TRPC6 channels was markedly attenuated in the podocytes that were treated with TRPC6 siRNA, whereas 1 μM 20-HETE increased currents in cells treated with control siRNA (Figures 1F,G). Moreover, using cell-surface biotinylation assays, we observed that exposure to 1 μM 20-HETE evoked an increase in steady-state surface expression of TRPC6 subunits (Figure 1H). Collectively these data indicate that 20-HETE is capable of modulating endogenously expressed TRPC6 channels of podocytes. We have previously shown that currents with similar properties are activated by exposing podocytes to a membrane-permeable diacylglycerol analog (OAG) (Anderson et al., 2013; Kim et al., 2013). Here we observed that 100 μM OAG evoked an additional increase in currents in podocytes after prior activation of TRPC6 by 20 μM 20-HETE. The current observed in the presence of both lipids was abolished by 50 μM La^3+^ (Figures 2A,B). This pattern was seen regardless of which lipid was applied first. Thus, superfusion with 20 μM 20-HETE increased current amplitudes even when it was applied after a steady-state response to 100 μM OAG was achieved (Figures 2C,D). Application of 50 μM La^3+^ abolished the currents seen in presence of both lipids, regardless of which lipid was applied first.

### 20-HETE effects on podocyte TRPC6 channels require ROS, G protein activation, and podocin

**Figure 3:**
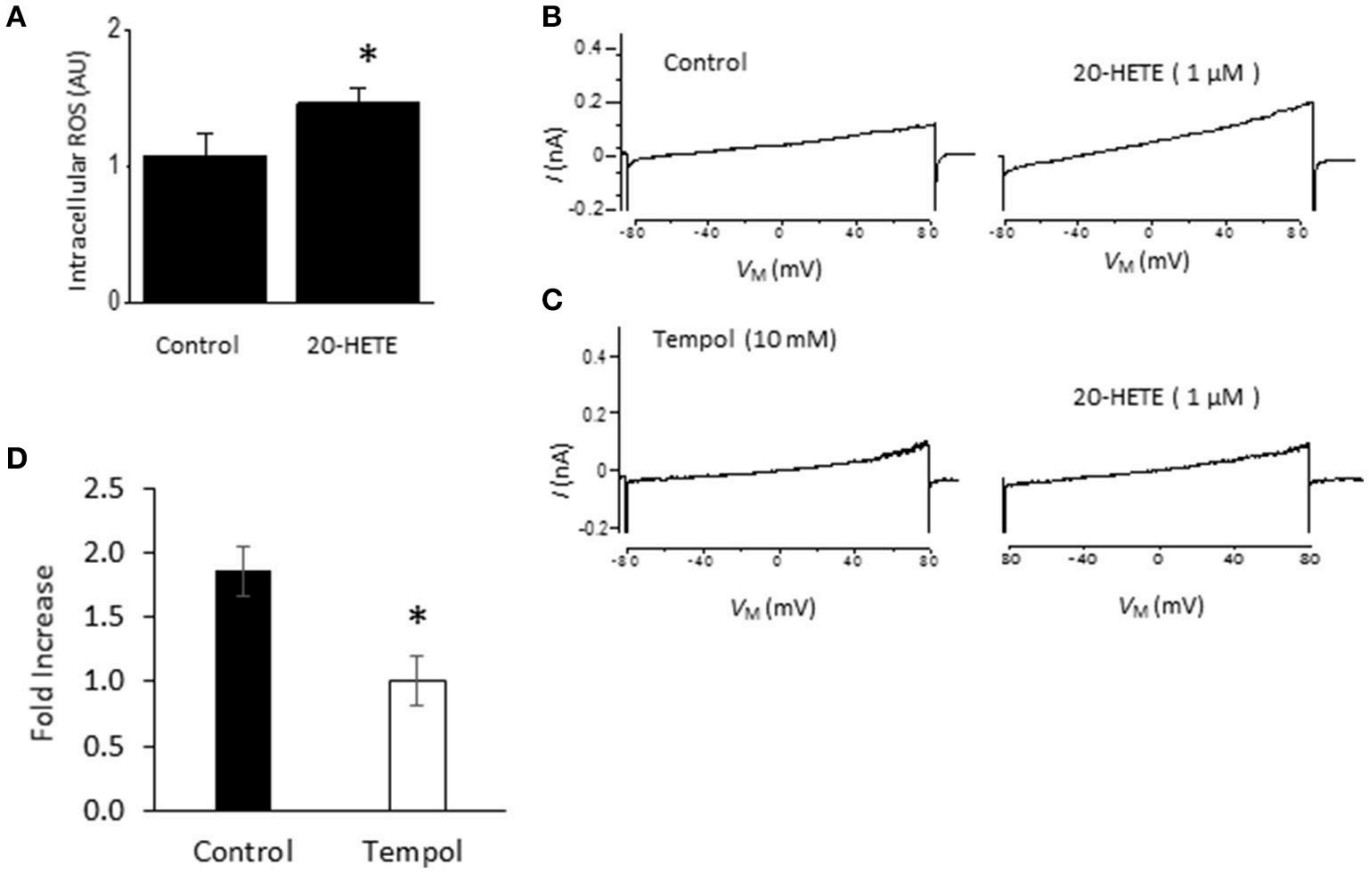
**Role of ROS in actions of 20-HETE on TRPC6. (A)** Increased bulk cytosolic ROS concentration in podocytes as measured using fluorescence assay in control cells and cells exposed to 1 μM 20-HETE for 24 h. Asterisk indicates *P* < 0.01 (unpaired *t*-test). **(B)** Traces showing responses to 1 μM 20-HETE. **(C)** Example of response to 1 μM 20-HETE in cell that had been pre-treated with 10 mM TEMPOL, an agent that quenches ROS. **(D)** Summary of the results of this experiment (*n* = 6 cells per group). Note complete inhibition of response to 20-HETE in cells pretreated with TEMPOL (asterisk indicates *P* < 0.05, unpaired *t*-test).

**Figure 4:**
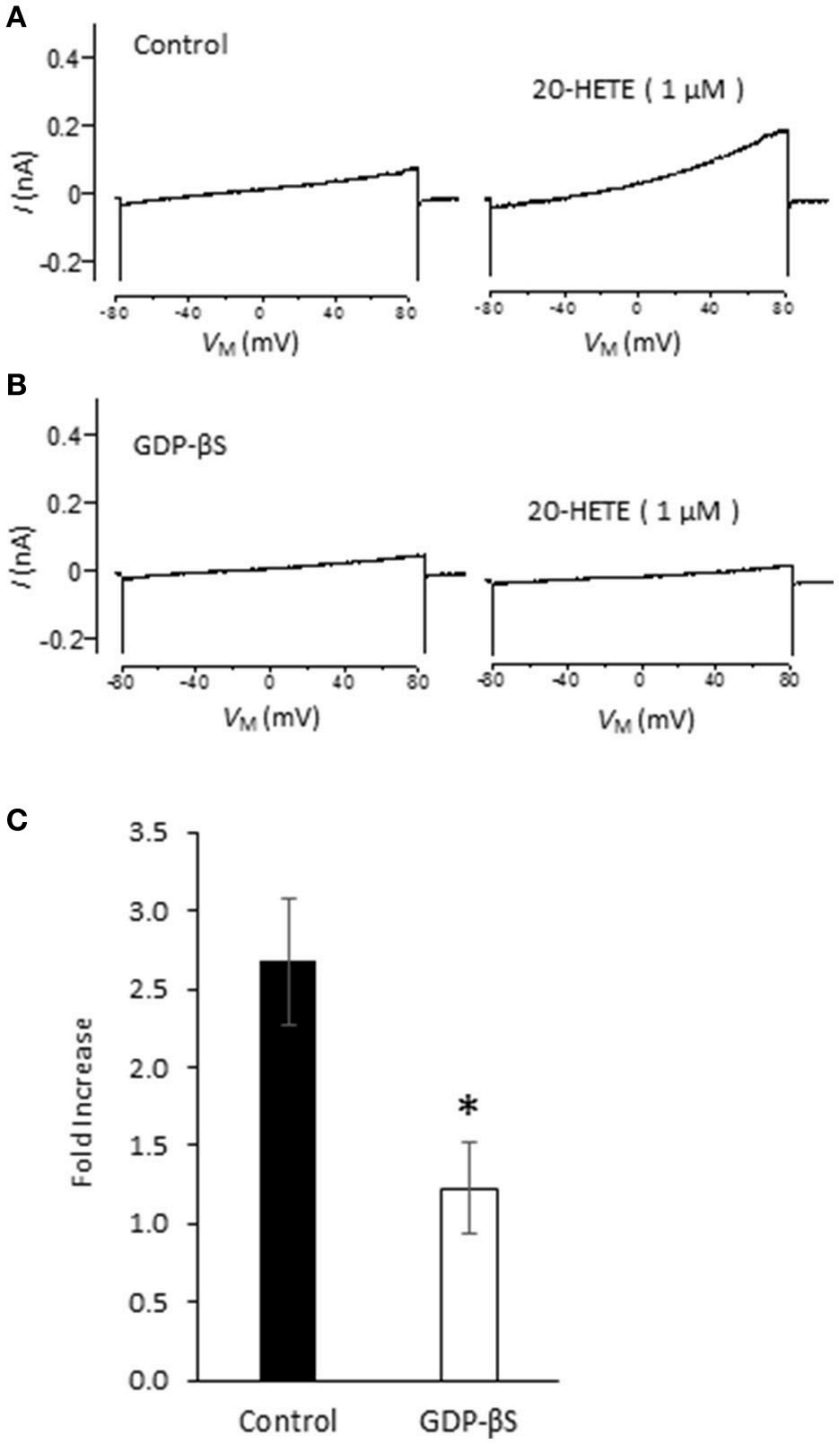
**G protein signaling is required for 20-HETE modulation of podocyte TRPC6 channels. (A)** Trace showing increased current evoked by 1 μM 20-HETE recorded with normal pipette saline. **(B)** Recording from different cell made with pipette containing 50 μM GDP-βS. Note that 20-HETE does not evoke a change in current under those conditions. **(C)** Summary of fold increase in current at +80 mV evoked by 20-HETE compared to baseline in absence or presence of GDP-βS in recording pipette. Asterisk indicates *P* < 0.05 (unpaired *t*-test, *n* = 15 cells per group).

Studies from our laboratory and from several others have shown that activation of podocyte TRPC6 channels by a variety of modulators requires generation of ROS (Kim et al., 2012a,b, 2013, 2015; Liu et al., 2013, 2015; Anderson et al., 2014; Ilatovskaya et al., 2014; Ma et al., 2016). Using a fluorescence assay, we observed that exposure to 1 μM 20-HETE for 24 h caused an increase in the bulk cytosolic concentration of ROS (Figure 3A). In previous studies we have also shown that NADPH oxidases occur as part of a much larger complex that includes TRPC6 channels and podocin (Kim et al., 2013). Because ROS concentration will increase quite rapidly within nanometers of the sites where they are generated, it is likely that TRPC6 channels are exposed to relatively high concentrations of ROS shortly after the onset of 20-HETE application. Consistent with this, we observed that 1 μM 20-HETE was no longer able to activate currents in podocytes pre-treated with 10 mM TEMPOL, a membrane-permeable superoxide dismutase mimic that causes quenching of ROS (Wilcox, 2010) (Figures 3B–D). The actions of 20-HETE on podocyte TRPC6 channels also require activation of G proteins within the cell. This was ascertained in experiments in which recording pipettes were filled with 50 μM guanosine-5′-*O*-(2-thiodiphosphate) (GDP-βS), which rapidly diffuses into the cell and prevents activation of either heterotrimeric or small GTPases (Eckstein et al., 1979; Kwong et al., 1993). We observed that this procedure prevented activation of cationic currents by 1 μM 20-HETE, whereas normal responses to 20-HETE were seen in recordings made in the same sessions and cell preparations using normal pipette solutions (Figure 4).

**Figure 5:**
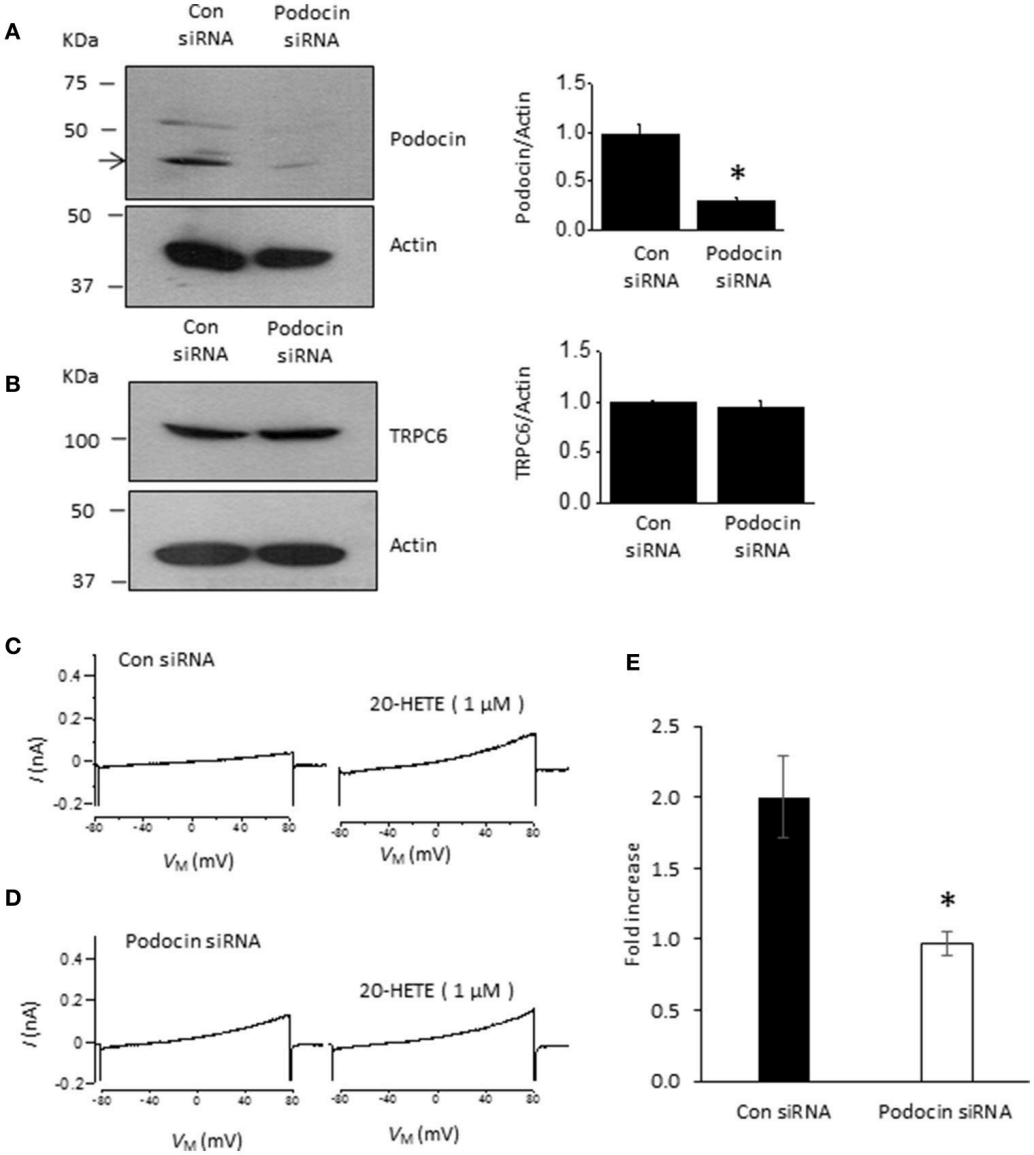
**Podocin is required for 20-HETE modulation of podocyte TRPC6 channels. (A)** Representative immunoblots showing podocin abundance in cells treated with non-targeting siRNA and siRNA targeting podocin. Bar graph summarizing three repetitions of this experiment is shown to the right. Asterisk indicates *P* < 0.01 (unpaired *t*-test). **(B)** Treatment with siRNA targeting podocin does not affect TRPC6 abundance. Representative immunoblots are shown to the left, and bar graph summarizing results from three different transfections is shown to the right. **(C)** Representative trace showing increased currents in podocyte evoked by 1 μM 20-HETE in cell treated with non-targeting siRNA. **(D)** Typical response to 1 μM 20-HETE in cell treated with siRNA targeting podocin. **(E)** Summary of fold increase in current at +80 mV evoked by 1 μM 20-HETE compared to baseline in cells treated with non-targeting siRNA (control) and siRNA targeting podocin. Asterisk indicates *P* < 0.05 (unpaired *t*-test, *n* = 6 cells per group).

Podocin organizes signaling complexes surrounding TRPC6 channels in podocyte foot processes. Therefore, we examined whether transient podocin knockdown using siRNA affects responses to 20-HETE (Figure 5). We observed that transfection with siRNA targeting podocin caused a reduction in podocin abundance compared to cells treated with non-targeting siRNA (Figure 5A), whereas total TRPC6 abundance was normal or close to normal in both groups (Figure 5B). As with our earlier studies on diacylglycerol analogs (Kim et al., 2013), we observed that activation of TRPC6 by 1 μM 20-HETE did not occur after podocin knockdown (Figures 5C–E).

## Discussion

Podocytes are terminally differentiated multipolar cells that cover the external surface of glomerular capillaries, and form an essential component of the glomerular filtration barrier. While it was once thought that podocytes and their slit diaphragms were simple static barriers, there is growing evidence that podocytes react and adapt to acute changes in glomerular filtration and hyperfiltration (Peti-Peterdi, 2006; Salmon et al., 2007). Podocytes are subjected to substantial mechanical forces owing to the net hydrodynamic filtration pressure, and shear forces produced by fluids flowing through slit diaphragms and the sub-podocyte space (Kriz et al., 2013; Kriz and Lemley, 2015). As a result, podocytes are at constant risk of detachment. Indeed, a podocytes tend to detach before dying, as viable podocytes can be detected in urine (Vogelmann et al., 2003). There is considerable evidence that loss of a certain threshold number of podocytes triggers a series of events that eventually leads to loss of the entire nephron (Kim et al., 2001; Kriz and LeHir, 2005; Macconi et al., 2006). Nevertheless, podocytes are normally able to maintain the stability of slit diaphragms in the face of large acute distending forces affecting the glomerular capillary (Kriz et al., 1994). The mechanisms whereby they accomplish this are complex and not well understood but certainly entail marked changes in cell cytoskeletal organization and morphology of foot processes. There is evidence that the foot process cytoskeleton is regulated in part by Ca^2+^-dynamics within podocytes (Tian et al., 2010; Vassiliadis et al., 2011). TRPC6 channels are major regulators of Ca^2+^ dynamics in podocytes (Dryer and Reiser, 2010; Tian et al., 2010), and normally occur at slit diaphragms in complex with other key slit diaphragm proteins (Reiser et al., 2005; Huber et al., 2006; Anderson et al., 2013). Moreover, there is now substantial evidence that while activation of TRPC6 channels may contribute acutely to normal physiological processes (Kim et al., 2012a), sustained and excessive activation of TRPC6 eventually leads to glomerular pathologies (Reiser et al., 2005; Winn et al., 2005; Krall et al., 2010; Wang et al., 2015). Consequently, it is important to identify physiologically occurring factors that can cause TRPC6 channels in podocytes to become active.

In the present study we show that 20-HETE increases current flowing through podocyte TRPC6 channels, as has been observed previously in vascular smooth muscle (Inoue et al., 2009). A more novel observation in this study is that the effects of 20-HETE on the TRPC6 channels of podocytes are indirect, as we observed that modulation of TRPC6 requires generation of ROS and activation of GTPases. Moreover, 20-HETE activation of TRPC6 was blocked by podocin knockdown. A substantial body of evidence now indicates that endogenously formed 20-HETE plays multiple roles in the regulation of renal function, especially within the pre-glomerular vasculature (Zou et al., 1994; Ge et al., 2014).

**Figure 6:**
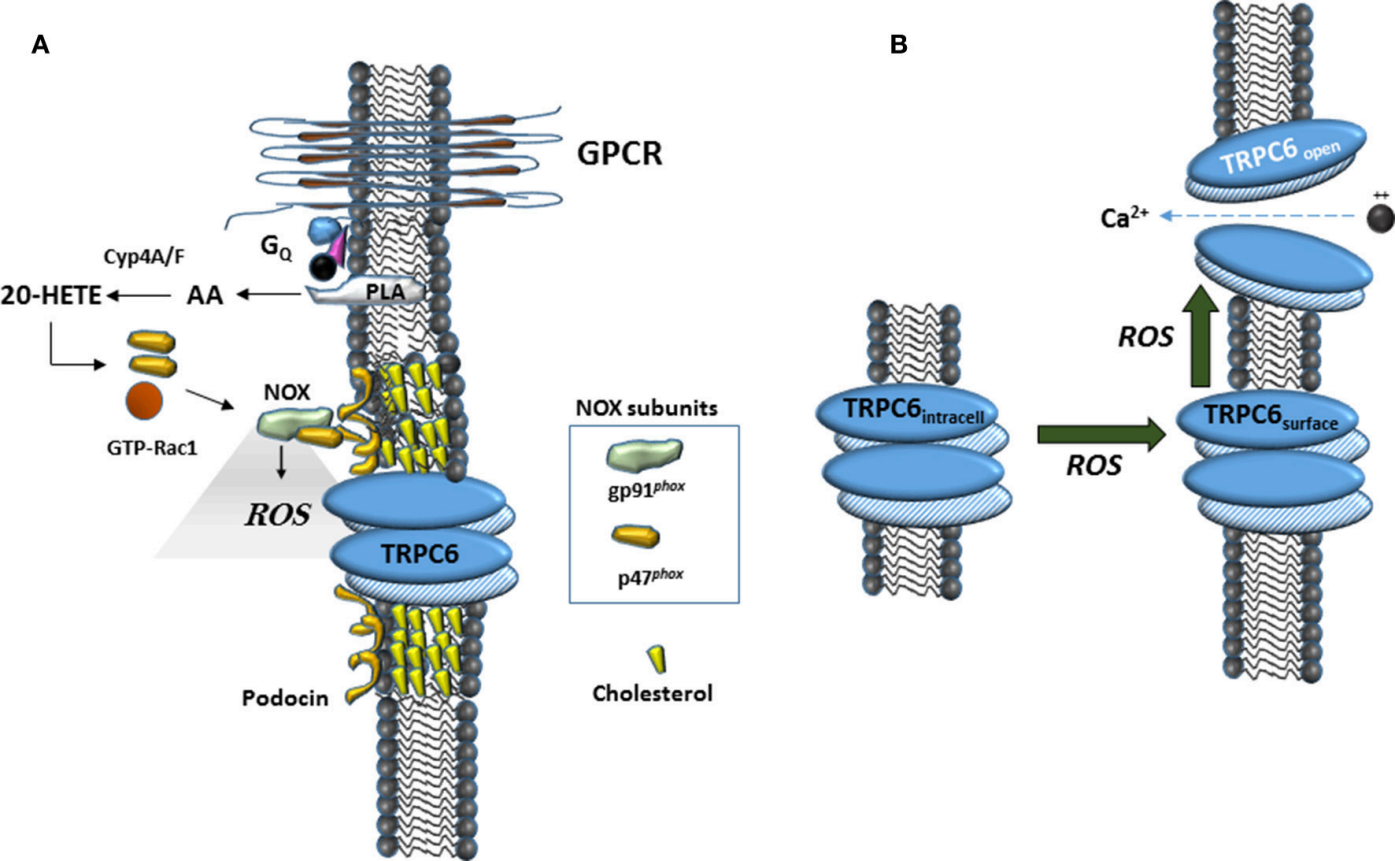
**Schematic diagrams suggesting mechanisms surrounding 20-HETE modulation of TRPC6 in podocytes. (A)** In many cell types, 20-HETE can be produced during G protein coupled signaling cascades, for example by G protein coupled receptor (GPCR) pathways activated by angiotensin II. In foot processes and in immortalized podocyte cell lines, a population of TRPC6 channels is part of a larger complex organized by podocin, a cholesterol-binding hairpin loop protein. In the presence of podocin, NOX enzymes are able to assemble in close proximity to TRPC6 channels, including for example formation of a ternary complex between gp91^*phox*^ and TRPC6 (Kim et al., 2013). This can lead to highly localized and rapid generation of ROS within the immediate vicinity of the channels. The effect of 20-HETE on podocyte TRPC6 channels requires G proteins, which may reflect a requirement of Rac activation for NOX2 or NOX4 activation (Bedard and Krause, 2007), or in other aspects of TRPC6 trafficking to the cell surface. **(B)** Data from many laboratories indicate that TRPC6 channels become active in presence of ROS, most likely though a combination of increased open probability of surface channels and increased steady-state localization at the cell surface.

In this study we have confirmed an earlier observation that 20-HETE increases the generation of ROS in podocytes (Eid et al., 2009). A similar effect has been observed in myocytes from cerebral arteries (Gebremedhin et al., 2014), as well as in cardiac myocytes where it occurs secondary to activation of NADPH oxidases (Zeng et al., 2010). Previous studies have shown that ROS is a powerful mediator of TRPC6 gating and trafficking (Graham et al., 2010; Ding et al., 2011) although it is notable that the role of ROS on TRPC6 appears to vary according to cell type (Graham et al., 2011; Bouron et al., 2016). The role of ROS in modulation of podocyte TRPC6 channels is summarized schematically in Figure 6. ROS generation appears to occur upstream of the actions of 20-HETE on TRPC6, as with DAG analogs, at least in this particular cell type (Kim et al., 2013). Previous studies have shown that ROS generation through NADPH oxidases is required for modulation of TRPC6 channels in podocytes in response to several different stimuli, including insulin (Kim et al., 2012a), angiotensin II (Anderson et al., 2014; Ilatovskaya et al., 2014), P2Y receptor agonists (Roshanravan and Dryer, 2014) and syndecans (Kim et al., 2015). The small GTPase Rac1 is required for activation of several NADPH oxidases, including NOX2 and NOX4 (Bedard and Krause, 2007). This by itself may suffice to explain why GDP-βS is also able to block TRPC6 activation by 20-HETE, although this procedure may affect other processes as well. In addition, the active form of NOX2 assembles as part of a complex with podocin and TRPC6 (Kim et al., 2013), and we have also detected interactions between podocin and NOX4 (unpublished data). These steps may be obligatory for any lipid-mediated transduction cascades that converge on TRPC6 channels in podocyte foot processes. As summarized in Figure 6, in the acute absence of podocin, formation of higher-order complexes that allow ROS generation in the immediate vicinity of TRPC6 channels are not possible. It is certainly possible that over time, some other member of the podocin/stomatin family of hairpin loop cholesterol binding proteins can take over this function.

Various diacylglycerols such as OAG can also activate TRPC6, indeed this was the first class of molecules ever shown to activate these channels (Hofmann et al., 1999). It is notable that the actions of relatively high concentrations of 20-HETE (at 20 μM) and OAG (at 100 μM) produced at least additive activation of endogenous podocyte TRPC6 channels, and the responses to these lipids do not occlude. This is consistent with previous studies in vascular smooth muscle, which have shown that 20-HETE enhances OAG-induced activation of TRPC6 channels in cells and excised patches (Inoue et al., 2009). In this regard, it is clear that 20-HETE and OAG can activate TRPC6 channels in a membrane-delimited fashion (Hofmann et al., 1999; Basora et al., 2003; Inoue et al., 2009). In podocytes this additive effect was seen even at the top of the dose-response curves for both lipids. This suggests that the mechanisms of the lipids diverge at some level, even though the actions of both lipids are dependent on podocin and ROS generation. The simplest possibility is that 20-HETE and OAG act on different subpopulations of TRPC6 within the cell. In this regard, we observed here that 20-HETE increased the steady-state surface expression of TRPC6 in podocytes. Previous studies have shown that rapid TRPC6 exocytosis also occurs during G_q_ mediated signaling, especially in cholesterol-rich membrane domains, and that this step preceded Ca^2+^ entry (Cayouette et al., 2004). Therefore, it is also possible that 20-HETE rapidly increases surface expression of TRPC6 channels which are then available for activation by OAG. Alternatively it is possible that the 20-HETE and OAG induce processes that act on different domains of the same TRPC6 channels. This later model is consistent with data obtained from vascular smooth muscle cells, in which 20-HETE and OAG appear to act at least additively in a membrane-delimited fashion in excised membrane patches (Inoue et al., 2009).

### Perspectives

Many studies on 20-HETE have framed their results in terms of a second-messenger type action for this lipid (Roman, 2002). However, there is now abundant evidence that 20-HETE can be stored and released, and can be excreted into urine, especially after exposure to angiotensin II (Carroll et al., 1997; Alonso-Galicia et al., 2002). Moreover, circulating 20-HETE appears to be increased in patients with atherosclerotic disease (Schuck et al., 2013). These observations raise the possibility that 20-HETE produces paracrine effects within glomeruli. As yet, no surface receptor for 20-HETE has been identified, and may not exist, but it is worth noting that other hydroxyeicosatetraenoic acids exert their effects in part through activation of specific G-protein coupled receptors, such as the OXE receptor and GPR31 (Powell and Rokach, 2015). Therefore, it remains possible that 20-HETE could cause modulation of podocyte TRPC6 channels through multiple mechanisms.

In general, 20-HETE has been considered renoprotective, in part because it is thought to reduce transmural pressure gradients across glomerular capillaries secondary to constriction of afferent arterioles (Fan et al., 2015). It clearly has actions in other renal cell types, and we show here that it causes activation of TRPC6 in podocytes. The physiological role of TRPC6 in podocytes is not fully understood but may play a role in allowing podocytes to acutely adapt to various mechanical and paracrine stimuli. In this regard, there is evidence that TRPC6 channels are acutely protective in an inflammatory milieu, for example during complement-mediated glomerular disease (Kistler et al., 2013). The underlying glomerular capillary pulsates, and there are substantial shear forces associated with hydrodynamic flow across the cell surface. The sub-podocyte space is also subjected to expansile forces. Collectively, these forces tend to promote detachment of podocytes. Bioactive agents such as angiotensin II and ATP can function as endocrine and/or paracrine signals and may lead to increased production of 20-HETE within podocytes, and it is possible that this plays an acute role in allowing podocytes to adapt to hyperfiltration. We have previously proposed that insulin actin in part through podocyte TRPC6 channels may also play a role in preparing glomeruli for hydrodynamic changes that occur as the kidney handles a glucose load (Kim et al., 2012a).

On the other hand, processes that are adaptive over short periods of time can become pathological if they are abnormally sustained. There are several lines of evidence that sustained activation of TRPC6 channels in podocytes contributes to foot process effacement and glomerulosclerosis (Reiser et al., 2005; Winn et al., 2005; Krall et al., 2010; Wang et al., 2015). There is also evidence that effacement itself, at least in the acute phase, is a mechanism that podocytes under stress use to prevent detachment (Kriz and Lemley, 2015). Given this, there may be conditions, for example during hyperglycemia or when glomerular hypertension is already well developed, when inhibition of 20-HETE synthesis or its actions on TRPC6 could be beneficial.

A limitation of this study is that analyses were carried out on a differentiated mouse podocyte cell line. This cell line has been widely used in the literature on experimental nephrology, and the cells express many of the key podocyte markers and regulatory molecules, including podocin, synaptopodin, and nephrin. However, their morphology is different from that of podocytes *in situ* which maintain contact with a glomerular capillary. It is therefore possible that certain signaling cascades no longer maintain their normal spatial relationships, and there are almost certainly differences in gene expression between the immortalized podocytes and native podocytes within glomeruli.

## Conclusions

We have shown that exogenous 20-HETE causes activation of podocyte TRPC6 channels. It is possible that this plays a role in regulation of glomerular function, for example during TGF, or during response to mediators such as angiotensin II. It may also play a role in pathological changes that occur in podocytes under various conditions.

## Author contributions

HR, EK, SD made substantial contributions to the conception and design of the work. HR and EK carried out the acquisition and analysis. HR, EK, and SD contributed to interpretation of data for the work. HR, EK, and SD drafted text and figures, and made revisions relative to important intellectual content. HR, EK, and SD all have final approval for this version of the work to be published. HR, EK, and SD agree to be accountable for all aspects of the work in ensuring that questions related to the accuracy or integrity of any part of the work are appropriately investigated and resolved.

## Funding

This research was supported by National Institutes of Health grant 1R01-DK104708 and initially by a contract from Pfizer Inc.

### Conflict of interest statement

Research in the corresponding author's laboratory was funded in part by a contract from Pfizer Inc. to study basic properties and regulation of TRPC6 in 2013–2014 administered through the Division of Research at the University of Houston. Other authors declare that the research was conducted in the absence of any commercial or financial relationships that could be construed as a potential conflict of interest.
